# Supplementary material for: Structural basis of lysophosphatidylserine receptor GPR174 ligand recognition and activation
Source: Nat Commun. 2023 Feb 23;14:1012. doi: 10.1038/s41467-023-36575-0 (PMC9950150; doi:10.1038/s41467-023-36575-0)
Supplement: Supplementary file 1 — Supplementary Information [file 41467_2023_36575_MOESM1_ESM.pdf]

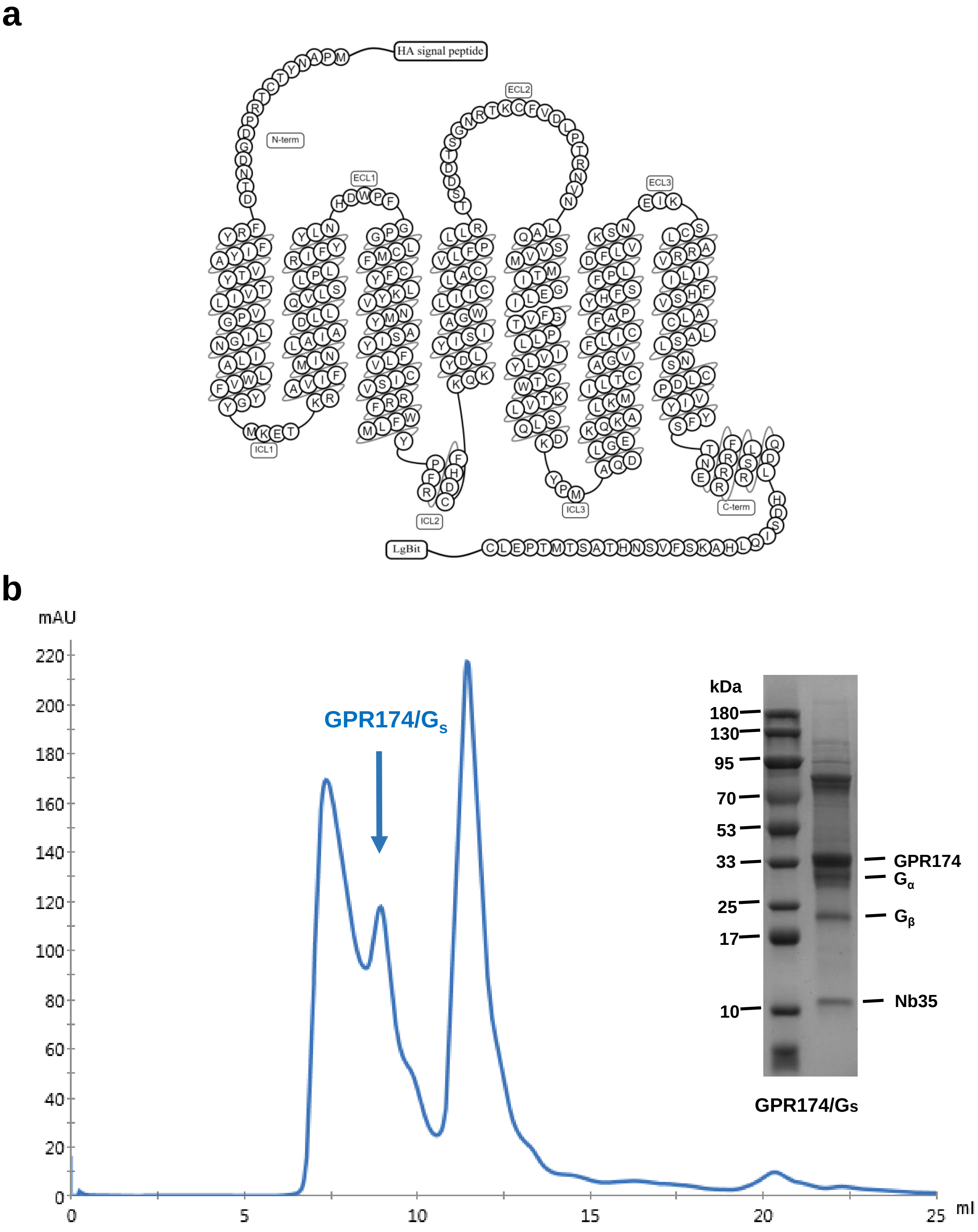

Supplementary Fig. 1. Expression and purification of GPR174. a, A snake-shape diagram of the mouse GPR174 construct used in complex assembling, the diagram was adopted from GPCRdb. **b**, Size exclusion column profile of GPR174/G_s complex. The experiment was repeated three times with similar results.

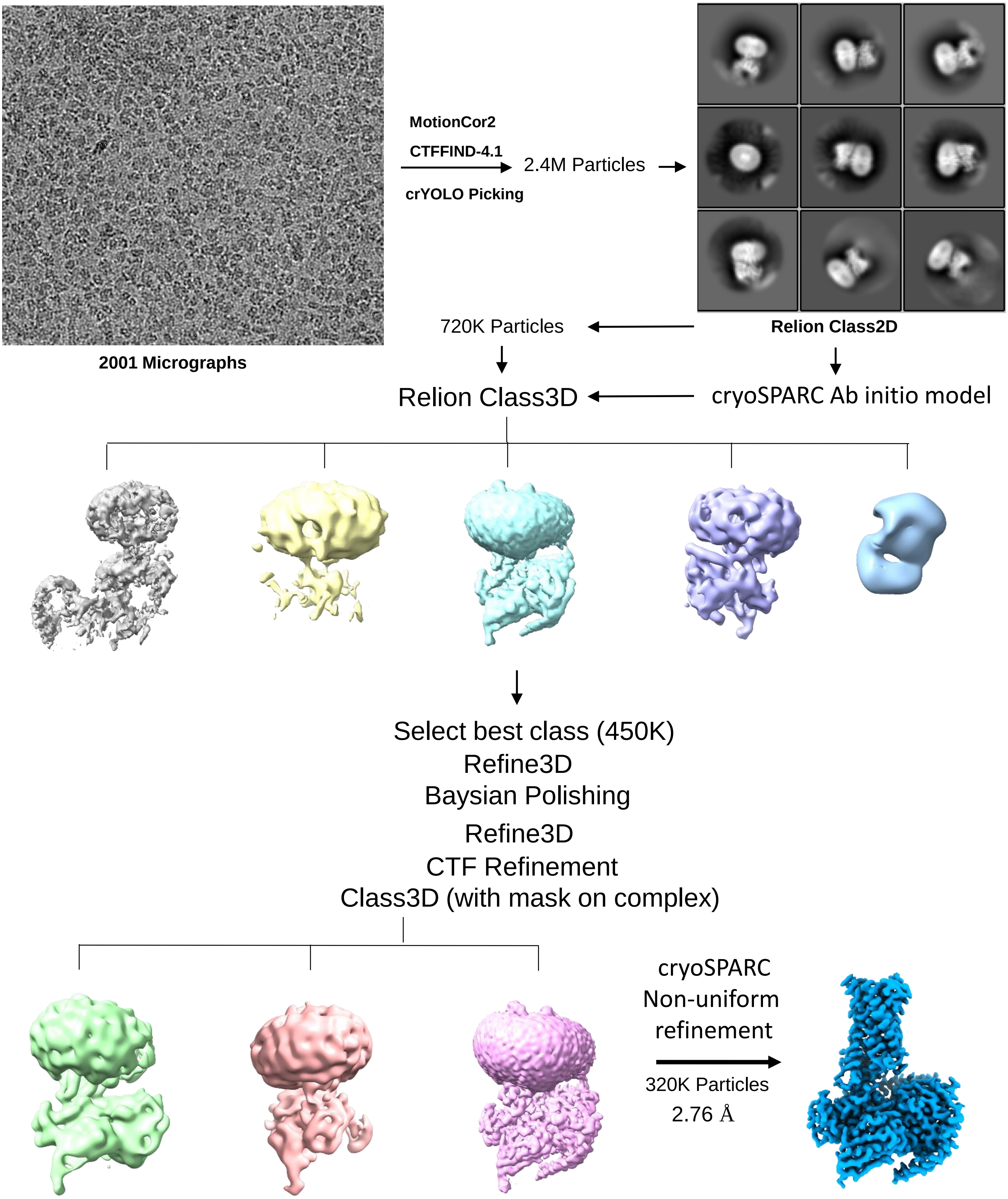

Supplementary Fig. 2. Flow-chart of cryo-EM data process of GPR174/G_s complex.

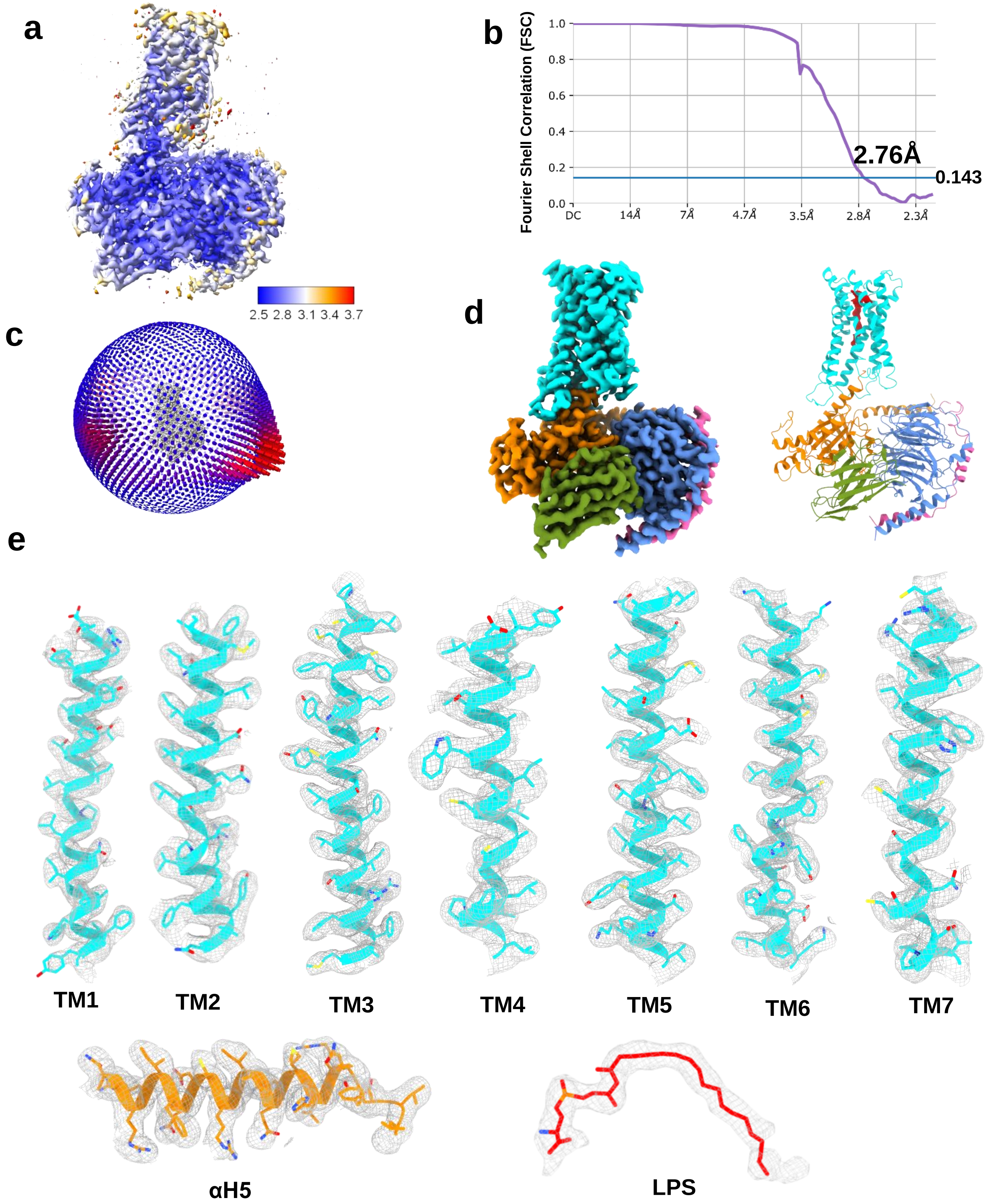

Supplementary Fig. 3. Resolution and representative map of GPR174/G_s complex. **a**, Local resolution analysis of GPR174/G_s complex. **b**, FSC curve of GPR174/G_s complex, the resolution was assessed by the Gold Standard of FSC=0.143. **c,** Angle distributions for particles contributing to the map of GPR174/G_s complex. **d**, A different viewing angle (comparing to Fig. 1) of GPR174/Gs map and model. **e**, Cryo-EM density map of representative regions of GPR174/G_s complex. Map level was set to 0.08 in chimera.

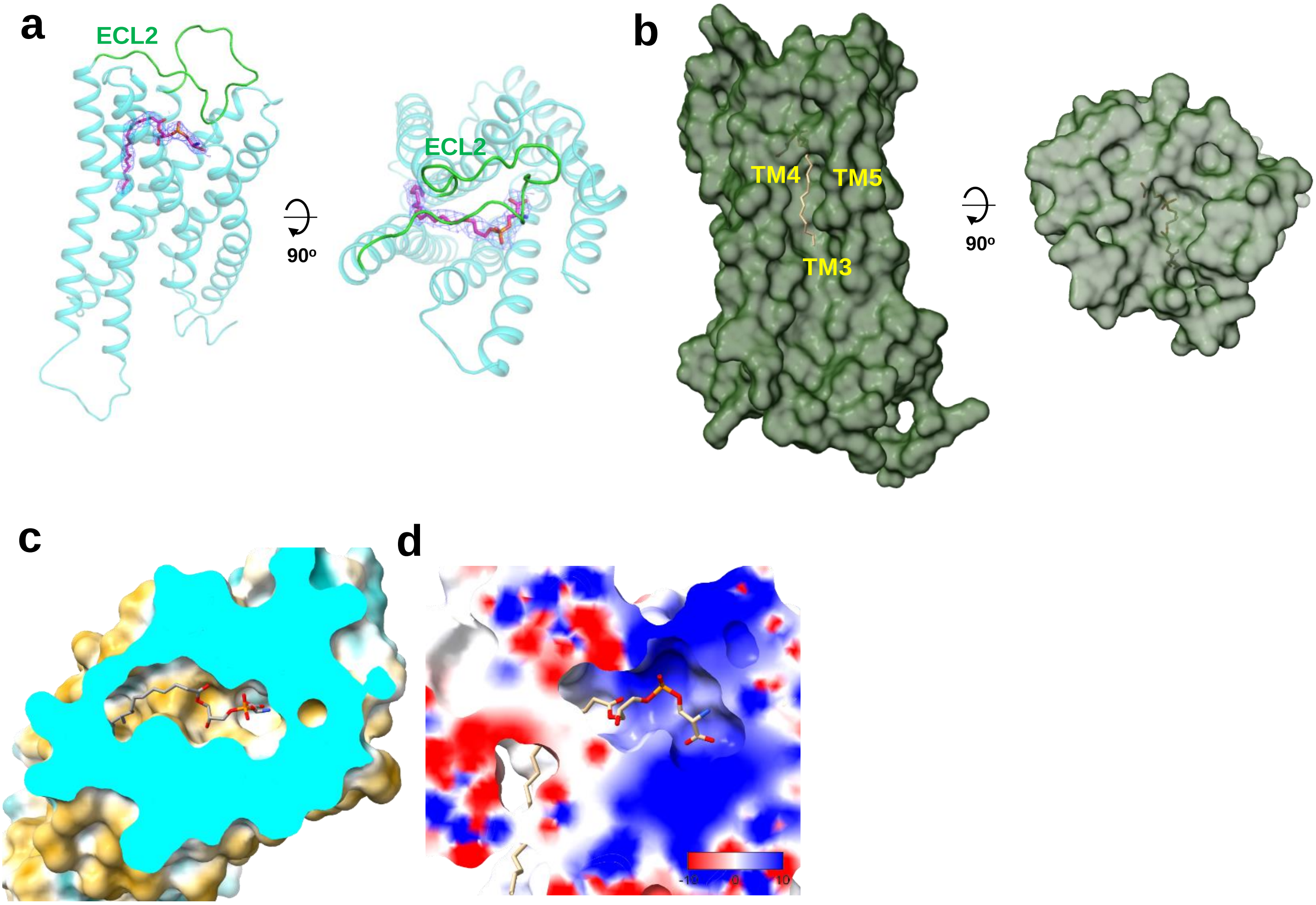

Supplementary Fig. 4. Additional information of the ligand binding pocket of GPR174. a, GPR174 has a long ECL2 over the ligand binding pocket. **b**, The ligand of LysoPS shown in surface model of the receptor. **c**, A hydrophobicity analysis of the ligand binding pocket of GPR174. **d**, An electrostatic analysis of the ligand binding pocket of GPR174.

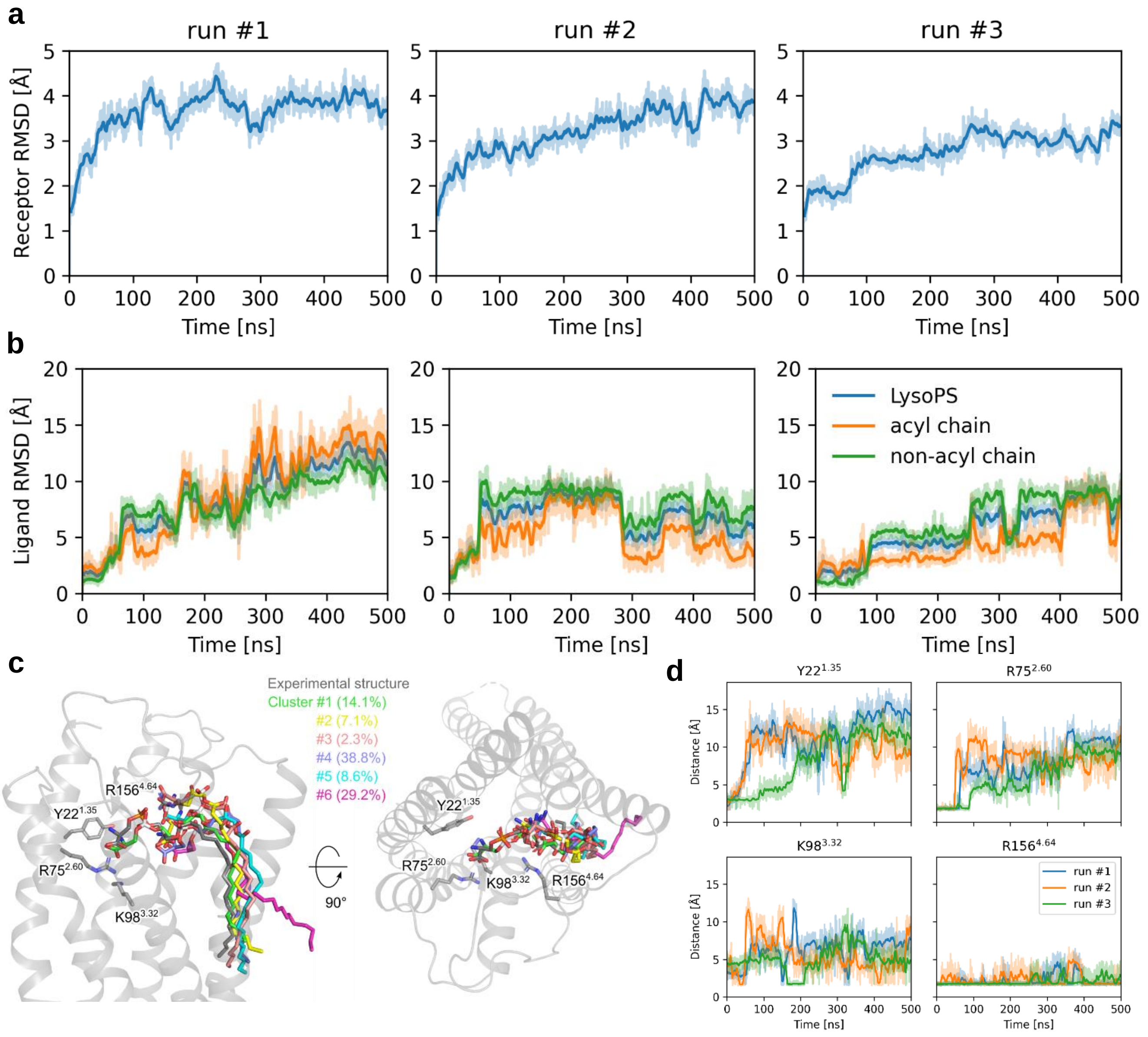

Supplementary Fig. 5. MD analysis of LPS ligand binding. a, RMSD traces of GPR174 Cα atoms during the simulations. Thick and thin traces indicates 5-ns moving averages and unsmoothed 100-ps traces, respectively. **b**, RMSD traces of heavy atoms in LysoPS during the simulations, calculated after aligning with GPR174. Thick and thin traces indicates 5-ns moving averages and unsmoothed 100-ps traces, respectively. **c**, Comparison with the experimental structure (gray) and representative structures (colored). **d**, Distance traces between LysoPS and sidechains identified in the structure and mutation study. Thick and thin traces indicates 5-ns moving averages and unsmoothed 100-ps traces, respectively.

a

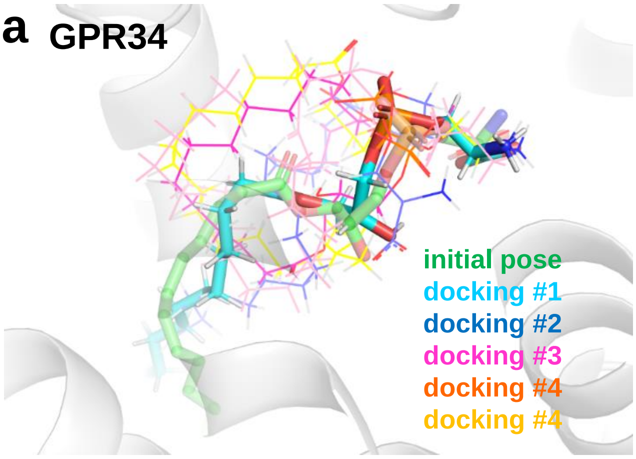

model #	S(ΔΔG)	rmsd(Å)
1	-10.69	1.9875
2	-10.2427	3.5763
3	-8.6728	6.4581
4	-8.609	8.5153
5	-8.4426	9.5497

b

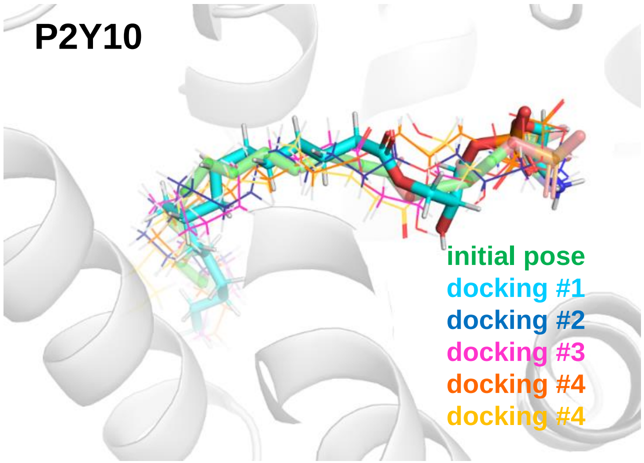

model #	S(ΔΔG)	rmsd(Å)
1	-10.9386	1.5451
2	-10.9161	2.3761
3	-10.831	1.9304
4	-10.3545	1.4424
5	-10.3494	2.3313

c

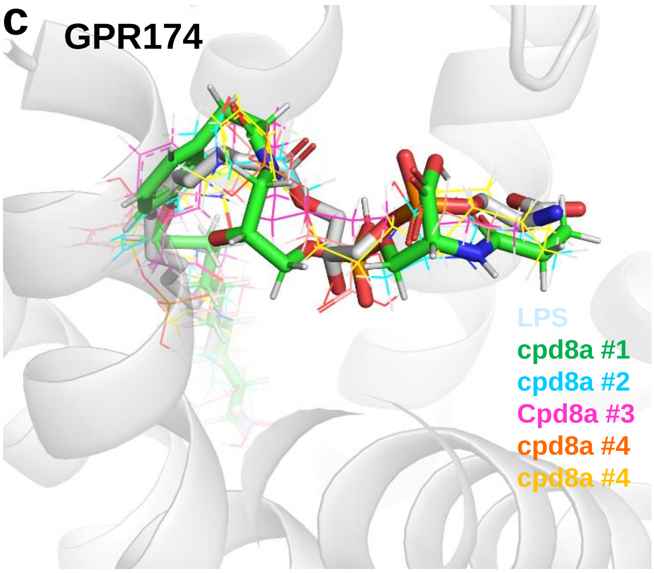

model #	S(ΔΔG)	rmsd_refine(Å)
1	-12.2224	2.8936
2	-11.7974	3.2201
3	-11.5329	2.3934
4	-11.4502	2.648
5	-11.2026	1.6189

d

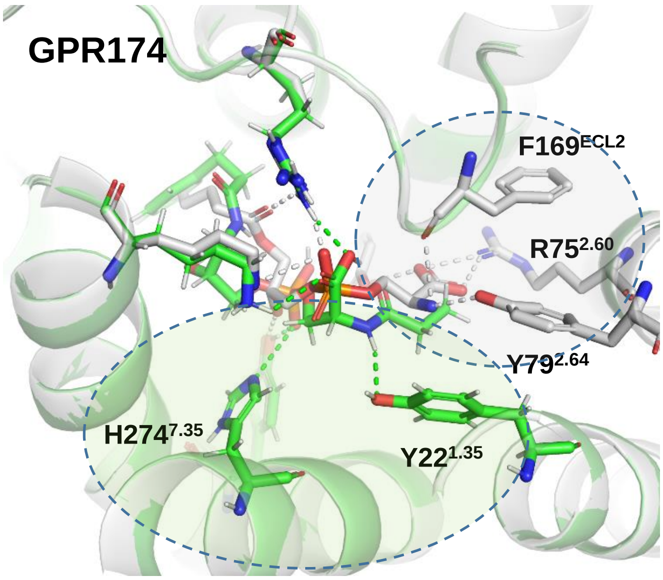

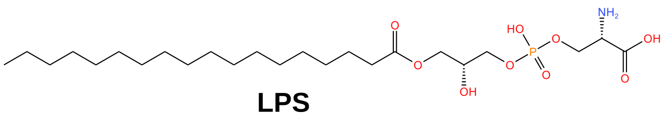

LPS

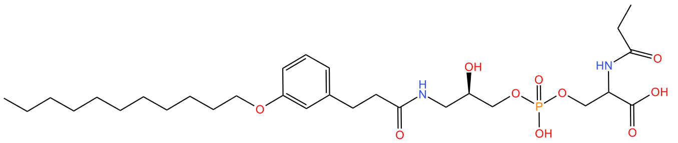

cpd8a

Supplementary Fig. 6. Docking analysis of GPR34, P2Y10 and GPR174. a, Docking of LysoPS into GPR34. **b**, Docking of LysoPS into P2Y10. **c**, Docking of N-acylated LysoPS compound, cpd8a, into GPR174. **d**, A detailed ligand/receptor interaction of the top scored cpd8a in GPR174.

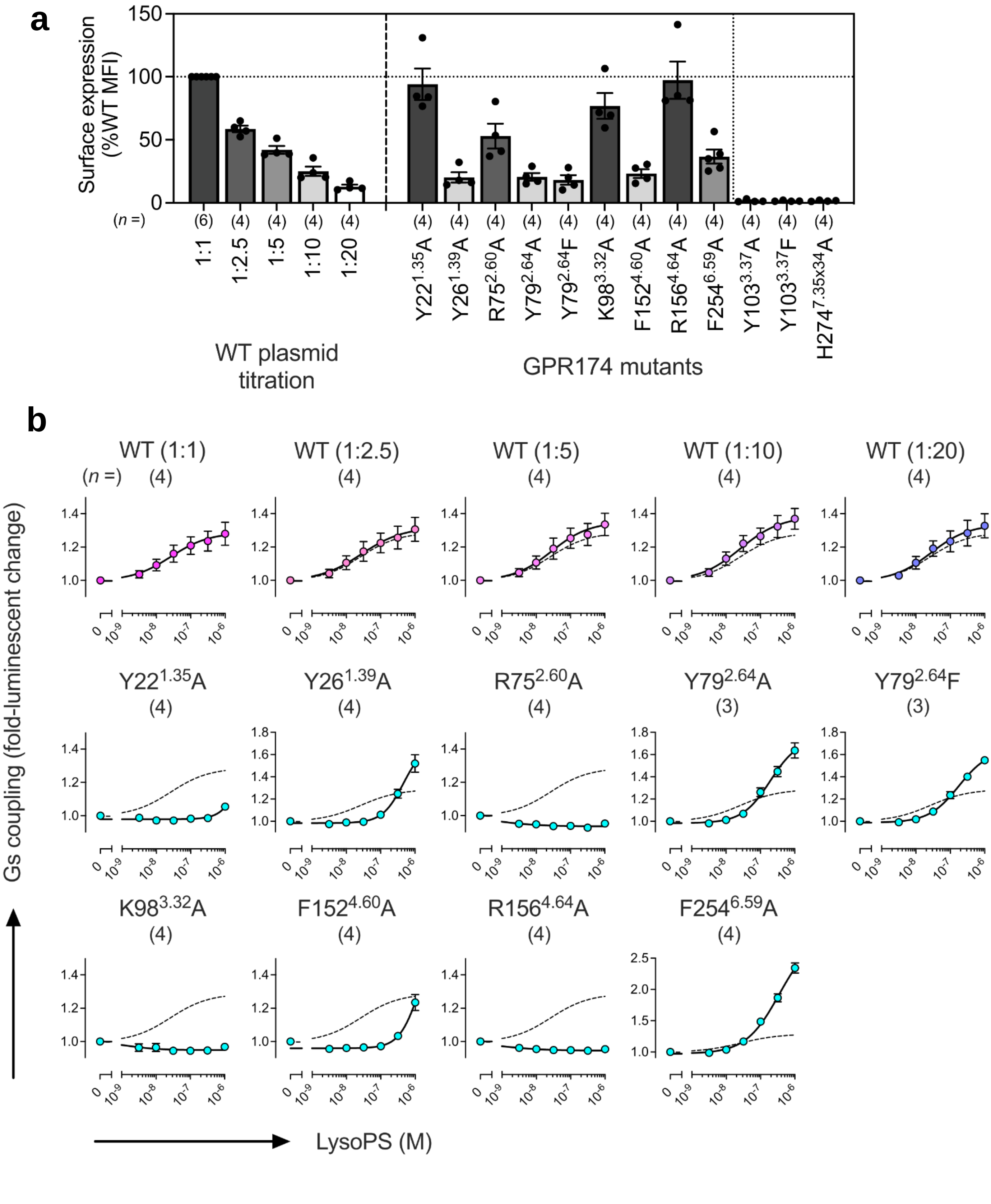

Supplementary Fig. 7. Functional assay of GPR174 mutants. a, Expression level of GPR174 mutants. Data were plotted as mean values ± SEM. Numbers in the parentheses below the x-axis denote numbers of independent experiments with individual data points shown as dots. **b**, NanoBrit assay of GPR174 mutants. Data are presented as mean values ± SEM. Numbers in the parentheses above the x-axis denote numbers of independent experiments with individual data points shown as dots. Dashed lines in each panel denote the WT (1:1) sigmoid concentration-response curve.

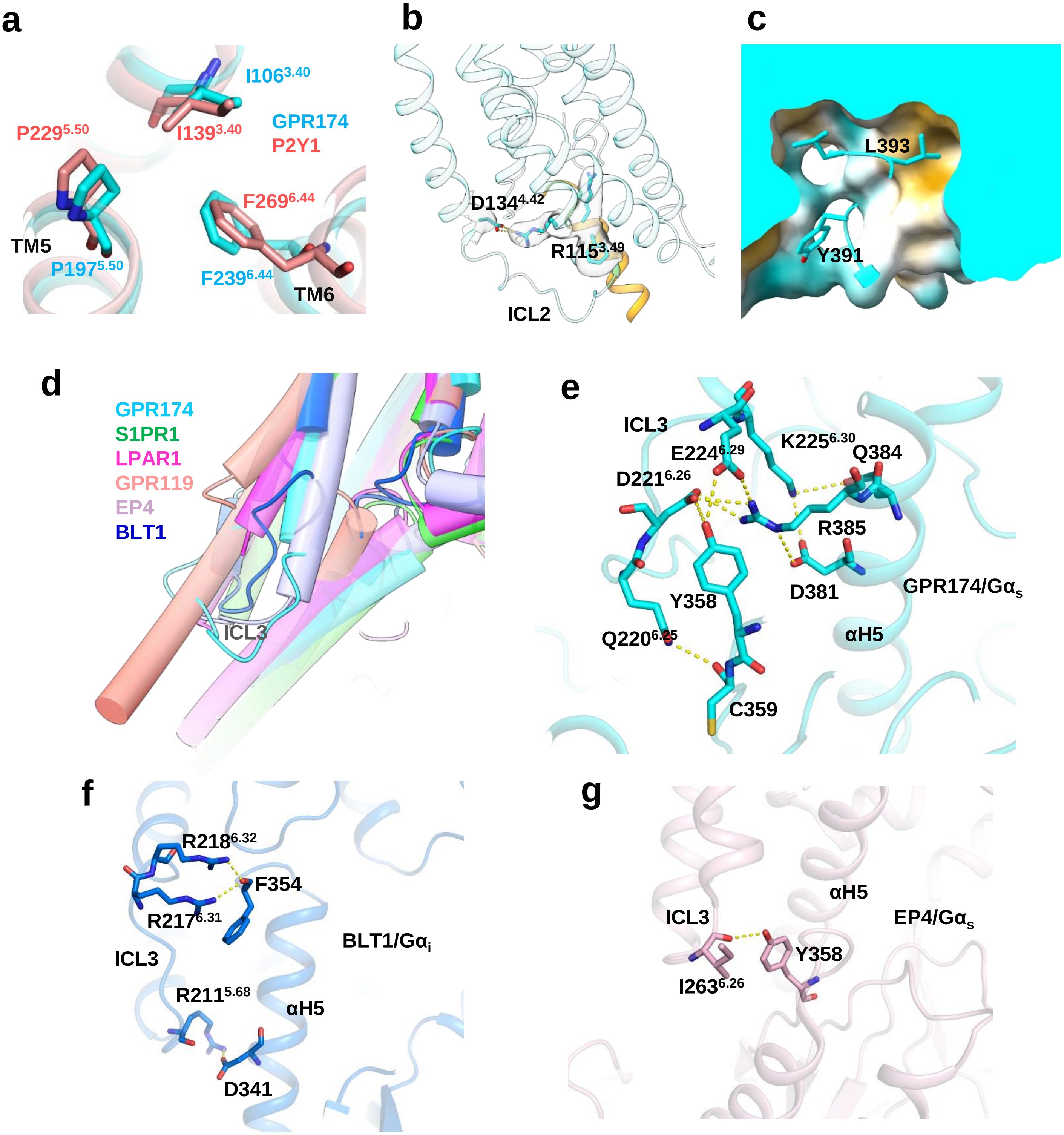

Supplementary Fig. 8. Additional information of GPR174 activation and G_s engagment. **a**, A comparison of the PIF motif between the active GPR174 and the inactive P2Y1 (PDB: 4xnw). **b**, R115$^{3.49}$ forms a salt bridge with D134$^{4.42}$ to locks and stabilizes ICL2. **c**, L393 of αH5 insert into a hydrophobic pocket in the intracellular cavity of GPR174. The yellow color of the surface indicates hydrophobicity. **d**, A comparison of ICL3 engagements of lipid receptors. **e**, Detailed ICL3 interaction in the GPR174/G_s complex. **f**, Detailed ICL3 interaction in the BLT1/G_i complex (PDB: 7vkt). **g**, Detailed ICL3 interaction in the EP4/G_s complex (PDB: 7d7m).

LysoPS

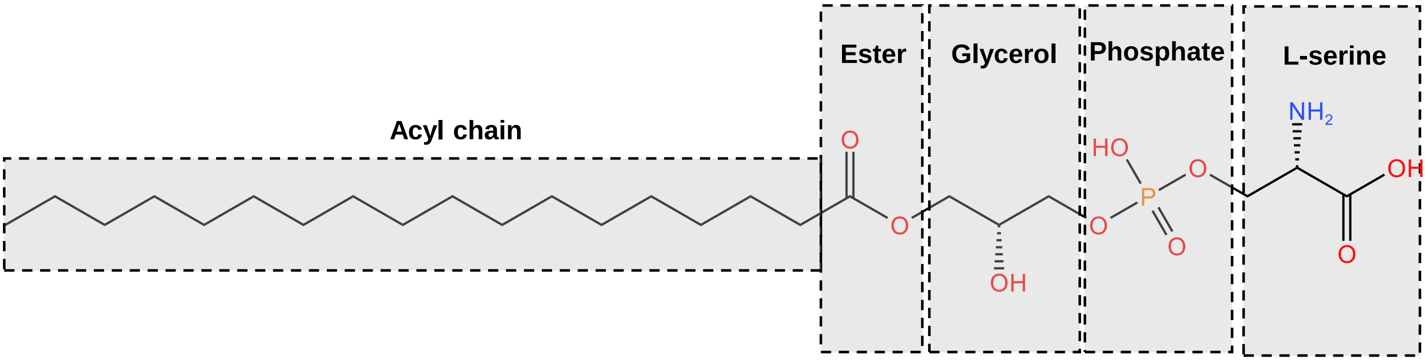

LPA

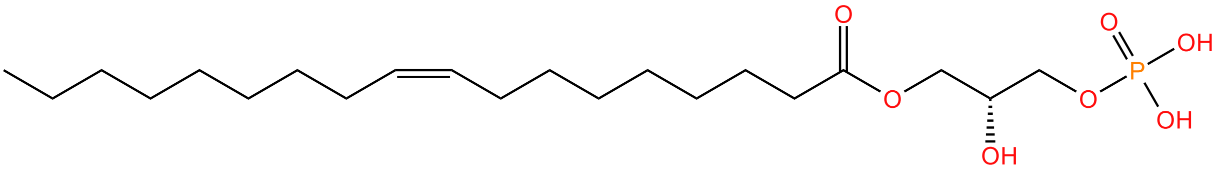

S1P

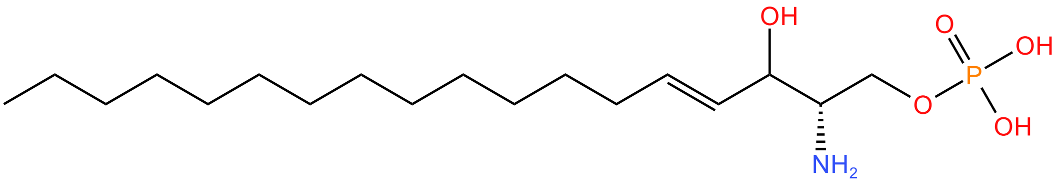

Supplementary Fig. 9. The chemical structures of LysoPS, LPA and S1P.

Supplementary Data Table 1| Cryo-EM data collection and refinement statistics

	GPR174/LPS/miniG$\alpha_s\beta\gamma$/Nb35<br>EMD-33479<br>7XV3
**Data collection and processing**	
Magnification	130,000
Voltage (kV)	300
Electron exposure (e^-/Å^2)	60
Defocus range (μm)	1.2-2.2
Pixel size (Å)	0.55
Symmetry imposed	C1
Initial particle image (no.)	2.4M
Final particle image (no.)	324k
Map resolution (Å)	2.76
FSC threshold	0.143
**Refinement**	
Initial model used (PDB code)	alpha-fold (AF-Q9BXC1), 7f4d
Model Resolution (Å)	3.3
FSC threshold	0.143
Map sharpening *B* factor (Å^2)	-115.4
Model composition	
Non-hydrogen atoms	8345
Protein residues	1045
Ligands	1
B factor (Å^2)	
Protein	47.6
Ligand	39.8
R.m.s. deviations	
Bond length (Å)	0.013
Bond angles (°)	1.505
Validation	
MolProbity score	1.18
Clashscore	0.97
Poor rotamers (%)	0
Ramachandran plot	
Favored (%)	94.19
Allowed (%)	5.81
Disallowed	0
