# Supplementary material for: Structural basis of lysophosphatidylserine receptor GPR174 ligand recognition and activation
Source: Nat Commun. 2023 Feb 23;14:1012. doi: 10.1038/s41467-023-36575-0 (PMC9950150; doi:10.1038/s41467-023-36575-0)
Supplement: Supplementary file 3 — Reporting Summary [file 41467_2023_36575_MOESM3_ESM.pdf]

nature portfolio

Corresponding author(s): Yuanzheng He

Last updated by author(s): Jan 27, 2023

Reporting Summary

Nature Portfolio wishes to improve the reproducibility of the work that we publish. This form provides structure for consistency and transparency in reporting. For further information on Nature Portfolio policies, see our Editorial Policies and the Editorial Policy Checklist.

Statistics

For all statistical analyses, confirm that the following items are present in the figure legend, table legend, main text, or Methods section.

n/a	Confirmed	
☐	☒	The exact sample size (*n*) for each experimental group/condition, given as a discrete number and unit of measurement
☐	☒	A statement on whether measurements were taken from distinct samples or whether the same sample was measured repeatedly
☐	☒	The statistical test(s) used AND whether they are one- or two-sided<br>*Only common tests should be described solely by name; describe more complex techniques in the Methods section.*
☒	☐	A description of all covariates tested
☒	☐	A description of any assumptions or corrections, such as tests of normality and adjustment for multiple comparisons
☐	☒	A full description of the statistical parameters including central tendency (e.g. means) or other basic estimates (e.g. regression coefficient) AND variation (e.g. standard deviation) or associated estimates of uncertainty (e.g. confidence intervals)
☐	☒	For null hypothesis testing, the test statistic (e.g. *F*, *t*, *r*) with confidence intervals, effect sizes, degrees of freedom and *P* value noted<br>*Give P values as exact values whenever suitable.*
☒	☐	For Bayesian analysis, information on the choice of priors and Markov chain Monte Carlo settings
☒	☐	For hierarchical and complex designs, identification of the appropriate level for tests and full reporting of outcomes
☒	☐	Estimates of effect sizes (e.g. Cohen's *d*, Pearson's *r*), indicating how they were calculated

Our web collection on statistics for biologists contains articles on many of the points above.

Software and code

Policy information about availability of computer code

Data collection	EPU-v2.2.0
Data analysis	Relion 3.1, CryoSPARC 2.15, Pymol 2.3.2, Coot 0.89, Phenix 1.18.2, MotionCor2.1, CTFFIND4.1, UCSF Chimera 1.15, crYOLO 1.7.4, MolProbity 4.1, LigPlot v2.2.5, modeller 9.25, 10.0, GROMACS 2020.4, Prism 9

For manuscripts utilizing custom algorithms or software that are central to the research but not yet described in published literature, software must be made available to editors and reviewers. We strongly encourage code deposition in a community repository (e.g. GitHub). See the Nature Portfolio guidelines for submitting code & software for further information.

Data

Policy information about availability of data

All manuscripts must include a data availability statement. This statement should provide the following information, where applicable:

- Accession codes, unique identifiers, or web links for publicly available datasets
- A description of any restrictions on data availability
- For clinical datasets or third party data, please ensure that the statement adheres to our policy

All data produced or analyzed in this study are included in the main text or the supplementary materials. The source data underlying Fig. 2d, and Supplementary Fig. S7a-b are provided as a Source Data file. A reporting summary for this article is available as a Supplementary Information file. The cryo-EM density maps and atomic coordinates have been deposited in the Electron Microscopy Data Bank (EMDB) and Protein Data Bank (PDB) under accession numbers EMD-33479 [https://

www.ebi.ac.uk/pdbe/entry/emdb/EMD-33479] and 7XV3 [http://doi.org/10.2210/pdb7XV3/pdb] for the GPR174/Gs complex. The mdp file for the MD simulation, topology file of LysoPS, and .pdb file for the system were deposited to Zenodo (10.5281/zenodo.7364300) [https://doi.org/10.5281/zenodo.7364300] and will be released upon the publication of the manuscript.

Human research participants

Policy information about studies involving human research participants and Sex and Gender in Research.

Reporting on sex and gender	not applicable
Population characteristics	not applicable
Recruitment	not applicable
Ethics oversight	not applicable

Note that full information on the approval of the study protocol must also be provided in the manuscript.

Field-specific reporting

Please select the one below that is the best fit for your research. If you are not sure, read the appropriate sections before making your selection.

☒ Life sciences ☐ Behavioural & social sciences ☐ Ecological, evolutionary & environmental sciences

For a reference copy of the document with all sections, see nature.com/documents/nr-reporting-summary-flat.pdf

Life sciences study design

All studies must disclose on these points even when the disclosure is negative.

Sample size	For all reporter functional assays, based on previous similar study (PMID: 36127364,36309016,35241677), three independent experiments (n=3-5) are carried out and sufficient. See figure legends for detail.
Data exclusions	No data were excluded
Replication	all experiments were repeated at least two times independently, all attempts at replication were successful.
Randomization	No Randomization was attempted or needed. Randomization was not necessary as the independent variables to be tested were sufficient for functional interpretation within this study. This is not a clinical trial or animal study that is dependent on randomization.
Blinding	Blinding was not necessary for structural determination as in this case, cryo-EM captured conformations representing a large amount of individual particles, or most time the major classes of all particles. Also, blinding is not necessary for functional analysis of this study as in this case, each mutations has it own distinct space coordinates. All experimental data acquired in this study are subjected to statistical analysis when necessary.

Reporting for specific materials, systems and methods

We require information from authors about some types of materials, experimental systems and methods used in many studies. Here, indicate whether each material, system or method listed is relevant to your study. If you are not sure if a list item applies to your research, read the appropriate section before selecting a response.

Materials & experimental systems

n/a	Involved in the study	
☐	☒	Antibodies
☐	☒	Eukaryotic cell lines
☒	☐	Palaeontology and archaeology
☒	☐	Animals and other organisms
☒	☐	Clinical data
☒	☐	Dual use research of concern

Methods

n/a	Involved in the study	
☒	☐	ChIP-seq
☒	☐	Flow cytometry
☒	☐	MRI-based neuroimaging

Antibodies

Antibodies used	anti-FLAG epitope (DYKDDDDK) tag monoclonal antibody, FujiFilm Wako Pure Chemicals # 012-22384
Validation	The validation of the The antibodies were validated by the manufactures in their specific data sheets. Anti-DYKDDDDK gives a low signal (less than 1:200 of WT S1PR1, as indicated in the method section) in mock-transfected HEK293 cells.

Eukaryotic cell lines

Policy information about cell lines and Sex and Gender in Research

Cell line source(s)	sf9 cell line, Invitorgen cat#11496-015; HEK293A cells, Thermo Fisher Scientific # R70507
Authentication	Cells have not been authenticated after purchase
Mycoplasma contamination	All cell lines tested are negative for mycoplasma contamination
Commonly misidentified lines (See ICLAC register)	No commonly misidentified cell lines were used
